# Aortic aneurysm trends attributable to high body mass index over the period 1990–2021 and projections up to 2040

**DOI:** 10.1007/s40200-025-01665-x

**Published:** 2025-06-26

**Authors:** Chao Li, Shixiang Qiu, Xianqiao Huang, Xing Deng, Yunguo Liao, Ziyu Tang, Jiaqi Pu, Xin Wei

**Affiliations:** 1 Department of Interventional Radiology, Beijing Anzhen Nanchong Hospital of Capital Medical University & Nanchong Central Hospital, No.97 Renmin South Road, Shunqing District, Nanchong, 637000 Sichuan Province China; 2 Department of Hepatobiliary Surgery, Beijing Anzhen Nanchong Hospital of Capital Medical University & Nanchong Central Hospital, Nanchong, China

**Keywords:** Obesity, Aortic aneurysm, Global burden of disease, Disability-adjusted life Years (DALY), Bayesian age-period-cohort (BAPC)

## Abstract

**Background:**

Obesity is a significant health issue globally, which can exacerbate aortic aneurysm (AA) diseases. AA is a type of cardiac aneurysm. As notable members of emerging economies, the BRICS nations collectively account for nearly 40% of the world’s population and generate approximately 25% of global GDP. The health systems of the BRICS countries are an important part of the global health system. The health system situation of the BRICS countries can to a large extent reflect the overall situation of the world’s health system. Understanding the impact trends of obesity in the BRICS countries (Brazil, the Russian Federation, India, China, and South Africa) is crucial due to their unique economic conditions and social backgrounds.

**Methods:**

Utilizing data from the Global Burden of Disease (GBD) database from 1990 to 2021, we extracted data related to AA and focused on mortality and years of life lost attributable to high BMI. We selected five countries with diverse geographic locations, economic development levels, healthcare systems, and demographic profiles. Descriptive analysis, decomposition analysis, and forecasting analysis were conducted to evaluate the impact of high BMI on the disease burden of AA and to predict future trends.

**Results:**

From 1990 to 2021, all five countries experienced an increase in mortality rates attributable to high BMI for AAs. For instance, China’s mortality rate increased from 0.0099 per 100,000 population in 1990 to 0.0376 per 100,000 in 2021. The Russian Federation had the largest increase, from 0.3370 per 100,000 in 1990 to 0.7283 per 100,000 in 2021. The trends in DALYs were consistent with those of mortality rates. In China, the DALY rate increased from 0.2788 per 100,000 in 1990 to 0.7449 per 100,000 in 2021. The EAPC analysis indicated that population aging and epidemiological changes were the primary drivers behind the increasing burden of AA attributable to high BMI. The forecasting analysis suggests a sustained increase in mortality rates due to AA attributable to high BMI across all examined countries.

**Conclusion:**

The findings are crucial for developing targeted preventive measures to alleviate the burden of AA over the coming decades, especially against the backdrop of rapidly aging populations and rapidly changing lifestyles.

**Supplementary Information:**

The online version contains supplementary material available at 10.1007/s40200-025-01665-x.

## Introduction

Aortic aneurysm (AA), encompassing thoracic aortic aneurysm (TAA) and abdominal aortic aneurysm (AAA), are characterized by the localized, irreversible dilation of the entire aortic wall [1]. This severe condition significantly escalates the risk of aortic dissection and sudden death [2]. Often asymptomatic, AA can lead to sudden aortic rupture and death, with survival rates plummeting below 20.0% [3]. According to the U.S. Preventive Services Task Force, the prevalence of AAA in the general population aged 60 years or older ranges from 1.6 to 7.2% [4]. Globally, AA-related deaths are estimated to claim between 150,000 and 200,000 lives annually [3].

Among the affected population, AA predominantly affects middle-aged and older adults, with a notably higher prevalence in individuals aged 65 years and above [5]. As global populations age rapidly, especially in regions such as Europe, Asia, and the Americas, the elderly demographic is burgeoning, leading to a concomitant increase in AA-related mortality [1]. Furthermore, as a critical cardiovascular disease, AA is aggravated by traditional risk factors including smoking, hypertension, nonalcoholic fatty liver disease, atherosclerosis, hyperlipidemia, a family history of aneurysms, and hypernatremia [6–8]. These factors are projected to further exacerbate the global burden of AA, particularly in developing countries where access to advanced healthcare is limited [1].

Additionally, emerging evidence highlights the role of body mass index (BMI) in AA pathogenesis. A study involving over 12,000 men revealed an independent association between central obesity and AAA, with serum resistin levels showing a stronger correlation with aortic diameter than adiponectin, a biomarker closely linked to obesity [9]. A study in the field of obesity and the obesity paradox had found severe obesity is associated with an increased risk of developing AAA and contributes to higher perioperative mortality, largely due to its complications and endocrine effects [10]. A meta-analysis study indicates that the association between central obesity and AAA is positive [11]. Another meta-analysis encompassing 18 studies further established a nonlinear relationship between BMI and AAA risk [12]. Notably, higher BMI categories are associated with increased 30-day mortality following AA repair [13]. Therefore, examining the impact of elevated BMI on the disease burden of AA is crucial for a comprehensive assessment of the condition’s epidemiology and public health implications.

## Methods

### Data sources

This study utilized data from the Global Burden of Disease (GBD) database, which encompasses data from 204 countries and territories, 21 GBD regions, and 5 Socio-demographic Index (SDI) regions from 1990 to 2021. The database includes information on over 300 diseases and injuries, as well as 87 risk factors [14, 15]. Data sources include censuses, medical records, research publications, and official mortality statistics, allowing for the assessment of disease burden and risk factors globally [14, 15]. Mortality data were derived from vital registration records classified by the International Classification of Diseases (ICD) system or from verbal autopsies conducted through household mortality surveys [16]. For each health state, disability-adjusted life-years (DALYs) were calculated by summing years of life lost due to premature mortality (YLLs) and years lived with disability (YLDs), with standardized disability weights applied [17].

For this study, we utilized the Joinpoint software and the R language package. We extracted data related to AA, with a specific focus on the mortality and years of life lost attributable to high BMI. We selected “high BMI” from the list of risk factors and “aortic aneurysm” from the list of causes. The five BRICS nations are strategically distributed across distinct super-regions of the globe: Brazil represents Latin America and the Caribbean, situated in South America; Russia spans Eastern Europe and Central Asia, bridging the Eurasian landmass; India anchors South Asia with its vast population and cultural influence; China dominates East Asia as the world’s second-largest economy; and South Africa serves as a pivotal nation in Sub-Saharan Africa. Data were analyzed for five countries—Brazil, the Russian Federation, India, China, and South Africa—chosen for their diverse geographic locations, levels of economic development, health care systems, and demographic profiles, thereby enhancing the generalizability of our findings. The study aimed to evaluate the impact of high BMI on the disease burden of AA and to forecast future trends.

### Statistical analysis

#### Descriptive analysis

This study utilized the GBD global standard population for age standardization, aiming to eliminate the interference caused by differences in age structures among various countries in the comparison of disease burdens, and to ensure the scientificity and comparability of cross-national and cross-period analyses. Age-standardized rates (ASRs) per 100,000 population were calculated using the IHME Bayesian regression tool DisMod-MR to analyze trends in age-standardized mortality rates, DALYs, and YLLs for the five countries from 1990 to 2021 [18]. To further understand temporal trends and inform preventive strategies, we estimated the annual percentage change (EAPC) using the following linear model: Y = α + βX + ε, where Y = ln (incidence/mortality/DALY), X = calendar year, and ε = error term. The EAPC was calculated as 100 × (e^β^−1), and 95% confidence intervals (CIs) were derived using the linear model [19]. CI calculates the sampling error of parameters through linear regression, reflecting the statistical significance of the trend; UI integrates data missing, model parameters and structural errors through Bayesian models to generate the probability range of disease rates, reflecting the credibility of the overall estimation. The EAPC is a robust indicator for monitoring changes in disease patterns over time [20]. In addition, we examined the disease burden attributable to high BMI for AA in different age groups (ranging from 25 to 29 to 95 + years) in the five countries in 2021. Mortality rates, YLL rates, and DALY rates were standardized per 100,000 population to ensure comparability across countries and age groups.

#### Decomposition analysis

We employed the decomposition analysis method proposed by Das Gupta et al. to quantify the contributions of changes in age structure, epidemiological patterns, and population growth to the overall changes in deaths, DALYs and YLLs [21]. This approach enables us to determine whether the observed changes in the burden of AA attributable to high BMI were primarily driven by population dynamics or actual changes in disease risk [17, 22]. By providing a comprehensive understanding of the underlying drivers of burden changes, this method enhances our ability to interpret the observed trends. DALY (Disability-Adjusted Life Year) consists of YLL (Years of Life Lost) and YLD (Years Lived with Disability). Among them, YLD is calculated by multiplying the number of people with the disease by the disability weight (a 0–1 standardized value determined based on health surveys or expert consensus, reflecting the severity of the disease) and the average duration of the disease.

#### Forecasting analysis

To predict the future burden of AA attributable to high BMI over the next 20 years in the absence of interventions, we used the Bayesian Age-Period-Cohort (BAPC) model. This model incorporates nested Laplace approximations and provides probabilistic forecasts based on age, period, and cohort factors [23]. It is particularly useful for predicting future trends in the context of significant population changes. Compared with methods relying solely on sample statistics, the BAPC model offers greater flexibility in parameter selection and prior probability distributions, allowing for more robust predictions of age-stratified incidence and mortality rates [16, 24]. The Markov Chain Monte Carlo (MCMC) algorithm is utilized to simulate the age-specific distribution of future disease burden. Without prior information to reduce subjective influence, the model stability is verified through historical data fitting. The uncertainty interval for prediction is quantified by the 95% confidence interval of the posterior distribution, reflecting the joint probability distribution of age, period, and cohort effects. The model for the future prevalence rate of BMI remains unchanged.

## Results

### Overall trends of AA burden attributable to high BMI, 1990–2021

**Fig. 1:**
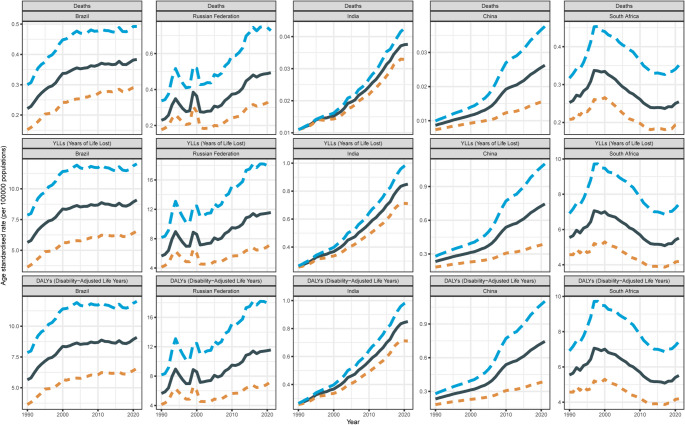
Overall trends of AA burden attributable to high BMI in five countries from 1990 to 2021

From 1990 to 2021, except for South Africa, another countries experienced an increase in mortality rates attributable to high BMI for AAs (Table 1; Fig. 1). The mortality rate in China increased from 0.0086 per 100,000 population in 1990 to 0.0262 per 100,000 in 2021. In Brazil, the rate rose from 0.2214 per 100,000 in 1990 to 0.3832 per 100,000 in 2021. India saw an increase from 0.0110 per 100,000 in 1990 to 0.0375 per 100,000 in 2021. The Russian Federation had the largest increase, from 0.2290 per 100,000 in 1990 to 0.4936 per 100,000 in 2021. South Africa began to rise rapidly after 0.2524 per 100,000 in 1990, reached a peak of 0.3345 per 100,000 in 2000, and then began to decline rapidly and reached a trough of 0.2389 per 100,000 in 2016. Then it showed an upward trend, and the mortality rate reached 0.2544 per 100,000 in 2021.

**Table 1:** ASDR and dalys of AA attributable to high BMI in five countries in 1990 and 2021, with EAPC from 1990 to 2021

		ASDR			Age-standardized DALY rate		
Country	Gender	1990 (95% UI)	2021 (95% UI)	EAPC (95% CI)	1990 (95% UI)	2021 (95% UI)	EAPC (95% CI)
China	Male	0.010 (0.005,0.019)	0.038 (0.019,0.067)	4.82 (4.58,5.06)	0.279 (0.128,0.541)	1.102 (0.564,2.021)	4.95 (4.7,5.2)
	Female	0.007 (0.003,0.015)	0.016 (0.007,0.029)	2.51 (2.44,2.59)	0.183 (0.080,0.380)	0.390 (0.179,0.714)	2.49 (2.41,2.57)
	Both	0.009 (0.004,0.016)	0.026 (0.013,0.047)	3.91 (3.73,4.08)	0.232 (0.115,0.4219)	0.745 (0.384,1.350)	4.11 (3.92,4.31)
Brazil	Male	0.300 (0.166,0.501)	0.492 (0.257,0.855)	1.28 (0.99,1.58)	7.859 (4.320,13.197)	12.026 (6.332,20.492)	0.98 (0.7,1.27)
	Female	0.152 (0.080,0.261)	0.293 (0.151,0.516)	1.85 (1.56,2.13)	3.633 (1.922,6.296)	6.557 (3.430,11.485)	1.49 (1.22,1.77)
	Both	0.221 (0.120,0.372)	0.383 (0.199,0.674)	1.47 (1.18,1.75)	5.646 (3.096,9.470)	9.091 (4.803,15.781)	1.14 (0.87,1.42)
India	Male	0.011 (0.004,0.026)	0.043 (0.018,0.086)	4.67 (4.57,4.78)	0.265 (0.096,0.639)	0.995 (0.4177,1.993)	4.46 (4.4,4.53)
	Female	0.011 (0.004,0.028)	0.033 (0.016,0.061)	3.84 (3.67,4.01)	0.257 (0.097,0.653)	0.711 (0.347,1.316)	3.54 (3.43,3.66)
	Both	0.011 (0.004,0.022)	0.038 (0.019,0.066)	4.26 (4.12,4.39)	0.261 (0.107,0.511)	0.850 (0.423,1.503)	4.03 (3.95,4.11)
South Africa	Male	0.317 (0.163,0.543)	0.351 (0.176,0.616)	-0.46 (-0.87,-0.06)	6.913 (3.517,11.934)	7.435 (3.761,13.231)	-0.56 (-0.97,-0.15)
	Female	0.208 (0.099,0.351)	0.195 (0.099,0.322)	-0.98 (-1.32,-0.65)	4.573 (2.322,7.697)	4.194 (2.145,6.971)	-0.87 (-1.11,-0.62)
	Both	0.252 (0.128,0.419)	0.254 (0.133,0.431)	-0.76 (-1.13,-0.4)	5.544 (2.844,9.327)	5.513 (2.892,9.251)	-0.72 (-1.04,-0.4)
Russian Federation	Male	0.337 (0.185,0.588)	0.728 (0.365,1.281)	2.38 (2,2.77)	8.167 (4.539,14.176)	17.624 (8.853,31.004)	2.35 (2.01,2.69)
	Female	0.177 (0.095,0.299)	0.343 (0.171,0.592)	1.55 (0.98,2.13)	4.129 (2.232,6.939)	7.247 (3.713,12.409)	1.13 (0.67,1.58)
	Both	0.229 (0.125,0.386)	0.494 (0.252,0.833)	2.16 (1.71,2.61)	5.642 (3.081,9.430)	11.562 (6.058,19.574)	1.95 (1.58,2.32)

The trends in DALYs were consistent with those of mortality rates (Table S1). In China, the DALY rate increased from 0.2323 per 100,000 in 1990 to 0.7449 per 100,000 in 2021. Brazil’s DALY rate rose from 5.6456 per 100,000 in 1990 to 9.0908 per 100,000 in 2021. India’s rate increased from 0.2612 per 100,000 in 1990 to 0.8499 per 100,000 in 2021. The Russian Federation had the largest increase, from 5.6415 per 100,000 in 1990 to 11.5617 per 100,000 in 2021. South Africa’s DALYs was largely consistent with changes in mortality trends, began to rise rapidly after 5.5439 per 100,000 in 1990, reached a peak of 7.0090 per 100,000 in 2000, and then began to decline rapidly and reached a trough of 5.083 per 100,000 in 2017. Then it showed an upward trend, and the mortality rate reached 5.5128 per 100,000 in 2021.

The trends in YLLs mirrored those of mortality and DALY rates (Table S1). In China, the YLL rate increased from 0.2323 per 100,000 in 1990 to 0.7449 per 100,000 in 2021. Brazil’s YLL rate rose from 5.6456 per 100,000 in 1990 to 9.0908 per 100,000 in 2021. India’s rate increased from 0.2612 per 100,000 in 1990 to 0.8500 per 100,000 in 2021. The Russian Federation had the most significant increase, from 5.6415 per 100,000 in 1990 to 11.5617 per 100,000 in 2021. Notably, South Africa’s YLLs was largely consistent with changes in mortality rate and DALYs, with a trend of rising first, then decreasing, and then rising slowly.

### EAPC of AA burden attributable to high BMI, 1990–2021

**Fig. 2:**
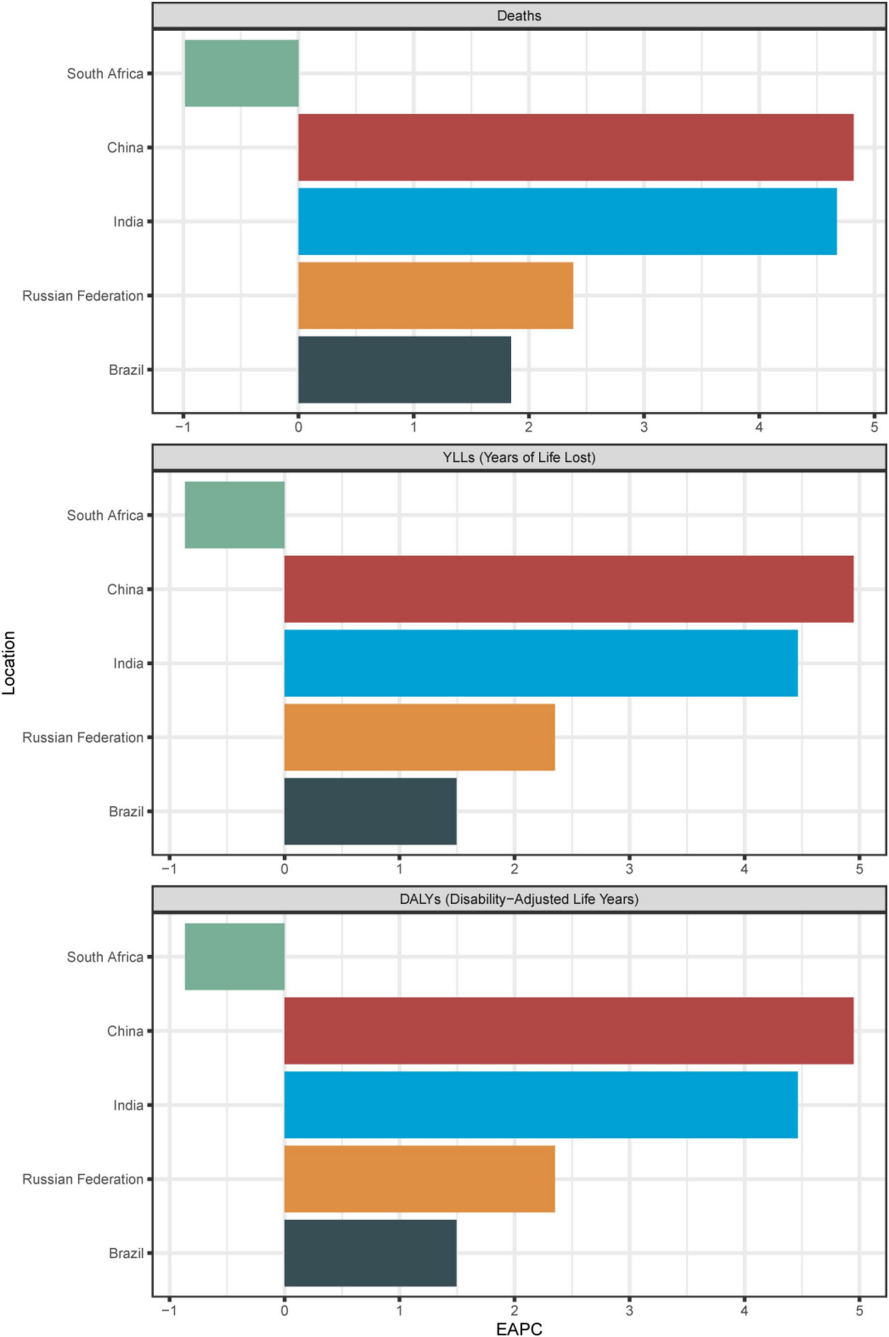
Bar chart for EAPC of AA burden attributable to high BMI in five countries from 1990 to 2021

Table S2 and Fig. 2 show the EAPC for AA burden attributable to high BMI in the five countries. For death, the EAPC for male mortality was 4.82 (95% CI, 4.58 to 5.06), for female mortality was 2.51 (95% CI, 2.44 to 2.59), and overall was 3.91 (95% CI, 3.73 to 4.08) in China. In Brazil, the EAPC for male mortality was 1.28 (95% CI, 0.99 to 1.58), for female mortality was 1.85 (95% CI, 1.56 to 2.13), and overall was 1.47 (95% CI, 1.18 to 1.75). In India, the EAPC for male mortality was 4.67 (95% CI, 4.57 to 4.78), for female mortality was 3.84 (95% CI, 3.67 to 4.01), and overall was 4.26 (95% CI, 4.12 to 4.39). In South Africa, the EAPC for male mortality was − 0.46 (95% CI, -0.87 to -0.06), for female mortality was − 0.98 (95% CI, -1.32 to -0.65), and overall was − 0.76 (95% CI, -1.13 to -0.40). In the Russian Federation, the EAPC for male mortality was 2.38 (95% CI, 2.00 to 2.77), for female mortality was 1.55 (95% CI, 0.98 to 2.13), and overall was 2.16 (95% CI, 1.71 to 2.61).

The EAPC of DALY rates showed similar trends. In China, the EAPC for male DALYs was 4.95 (95% CI, 4.70 to 5.20), for female DALYs was 2.49 (95% CI, 2.41 to 2.57), and overall was 4.11 (95% CI, 3.92 to 4.31). In Brazil, the EAPC for male DALYs was 0.98 (95% CI, 0.70 to 1.27), for female DALYs was 1.49 (95% CI, 1.22 to 1.77), and overall was 1.14 (95% CI, 0.87 to 1.42). In India, the EAPC for male DALYs was 4.46 (95% CI, 4.40 to 4.53), for female DALYs was 3.54 (95% CI, 3.43 to 3.66), and overall was 4.03 (95% CI, 3.95 to 4.11). In South Africa, the EAPC for male DALYs was − 0.56 (95% CI, -0.97 to -0.15), for female DALYs was − 0.87 (95% CI, -1.11 to -0.62), and overall was − 0.72 (95% CI, -1.04 to -0.40). In the Russian Federation, the EAPC for male DALYs was 2.35 (95% CI, 2.01 to 2.69), for female DALYs was 1.13 (95% CI, 0.67 to 1.58), and overall was 1.95 (95% CI, 1.58 to 2.32).

The EAPC of YLL rates was consistent with the trends observed in mortality and DALY rates. In China, the EAPC for male YLLs was 4.95 (95% CI, 4.70 to 5.20), for female YLLs was 2.49 (95% CI, 2.41 to 2.57), and overall was 4.11 (95% CI, 3.92 to 4.31). In Brazil, the EAPC for male YLLs was 0.98 (95% CI, 0.70 to 1.27), for female YLLs was 1.49 (95% CI, 1.22 to 1.77), and overall was 1.14 (95% CI, 0.87 to 1.42). In India, the EAPC for male YLLs was 4.46 (95% CI, 4.40 to 4.53), for female YLLs was 3.54 (95% CI, 3.43 to 3.66), and overall was 4.03 (95% CI, 3.95 to 4.11). In South Africa, the EAPC for male YLLs was − 0.56 (95% CI, -0.97 to -0.15), for female YLLs was − 0.87 (95% CI, -1.11 to -0.62), and overall was − 0.72 (95% CI, -1.04 to -0.40). In the Russian Federation, the EAPC for male YLLs was 2.35 (95% CI, 2.01 to 2.69), for female YLLs was 1.13 (95% CI, 0.67 to 1.58), and overall was 1.95 (95% CI, 1.58 to 2.32).

### Age distribution of AA burden attributable to high BMI in 2021

**Fig. 3:**
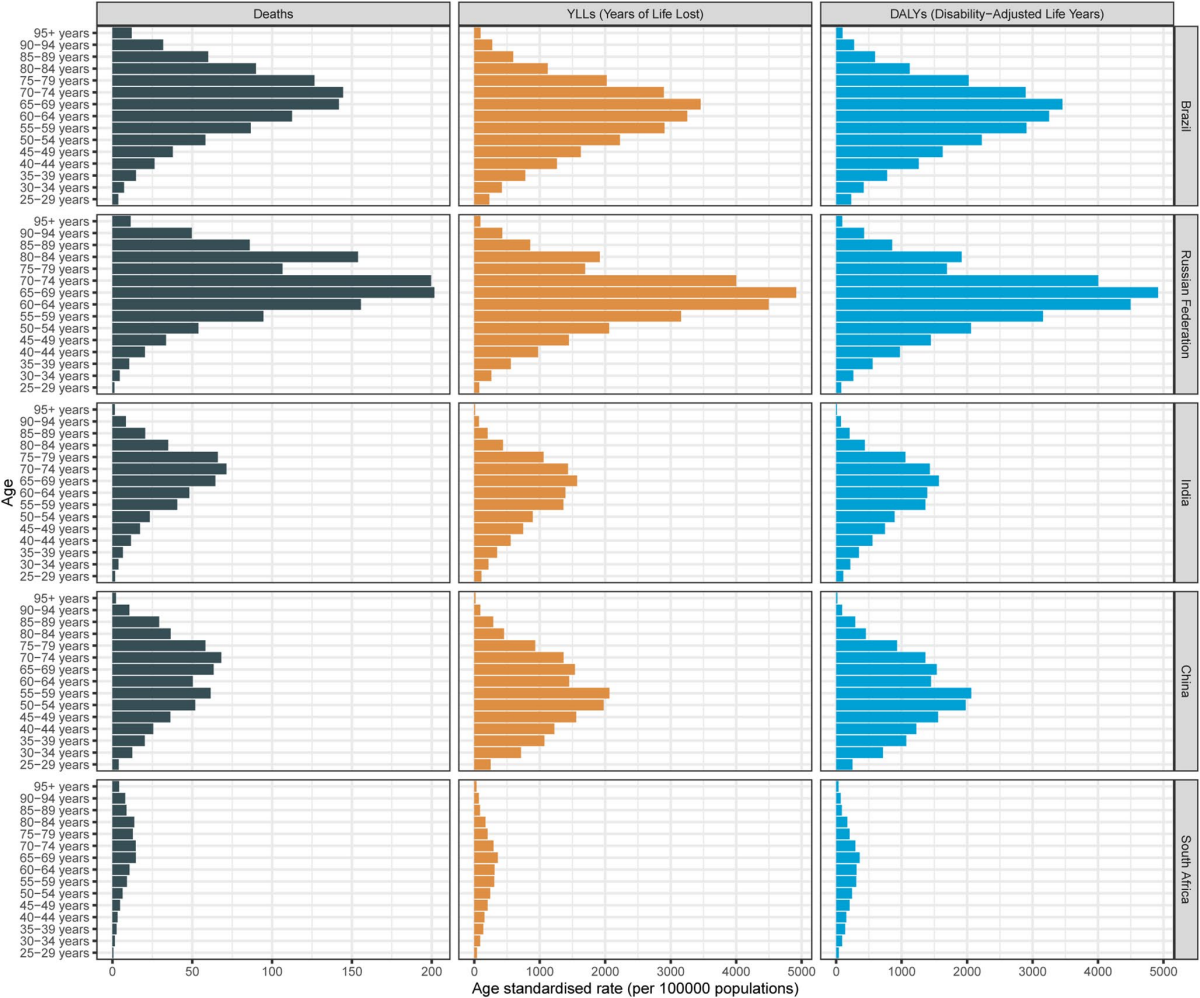
Age distribution of AA burden attributable to high BMI in five countries in 2021

Figure 3 shows the age distribution of AAs due to BMI in the five countries in 2021. Mortality rates increased significantly with age, peaking in individuals aged 60 years and older. China and India had the highest mortality rates in the oldest age groups (≥ 75–79 years), significantly higher than those in other countries. Brazil and South Africa had relatively lower mortality rates in middle-aged groups (40–64 years), but these rates also increased sharply in older age groups. The Russian Federation had intermediate mortality rates across all age groups, with a slight decline in the 70–74 years and older age groups.

The age distribution of DALYs further characterized the disease burden of AAs. DALY rates also increased significantly with age, peaking in individuals aged 70–74 years and older. China and India had the highest DALY rates in older age groups, indicating a more severe impact of AAs on the elderly in these countries. Brazil and South Africa had relatively lower DALY rates in middle-aged groups but showed a significant increase in older age groups. In contrast, the Russian Federation had intermediate DALY rates across all age groups, with a slight decline in the 70–74 years and older age groups.

The age distribution of years of YLLs mirrored the mortality rates, highlighting the significant impact of AAs on older populations. YLL rates were relatively low in younger age groups (25–29 to 45–49 years) but increased rapidly with age, peaking in individuals aged 60–64 years and older. Similar to mortality rates, China and India had the highest YLL rates in older age groups, while Brazil and South Africa had relatively lower YLL rates in middle-aged groups but showed a marked increase in older age groups.

### Decomposition analysis of AA burden attributable to high BMI

We conducted a decomposition analysis to further elucidate the contributions of population aging, population growth, and epidemiological changes to mortality and DALYs, YLLs due to high BMI for AA (Table S3).

**Fig. 4:**
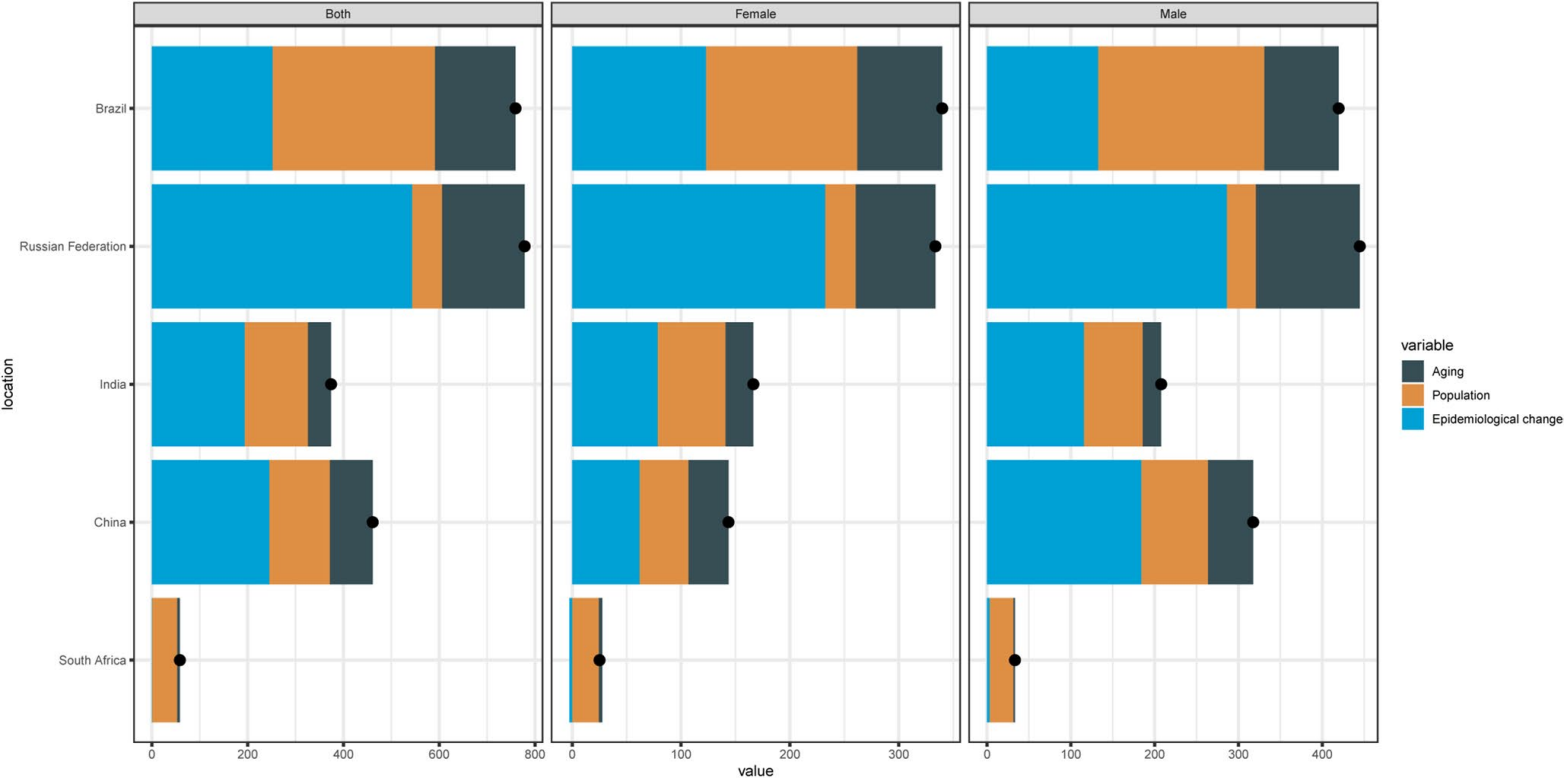
Contribution of population aging and epidemiological changes to mortality rate changes in five countries

The changes in mortality rates were primarily driven by population aging and epidemiological changes. For example, in Brazil, population aging and epidemiological changes contributed 21.2% and 31.6%, respectively, to the mortality rate changes in men. In India, epidemiological changes accounted for 55.6% of the mortality rate changes in men and 47.2% in women. In China and the Russian Federation, epidemiological changes contributed significantly to mortality rate changes, reaching 57.9% and 64.3%, respectively. In contrast, population growth was the major contributor to mortality rate changes in South Africa, accounting for 85.1% **(**Fig. 4**)**.

**Fig. 5:**
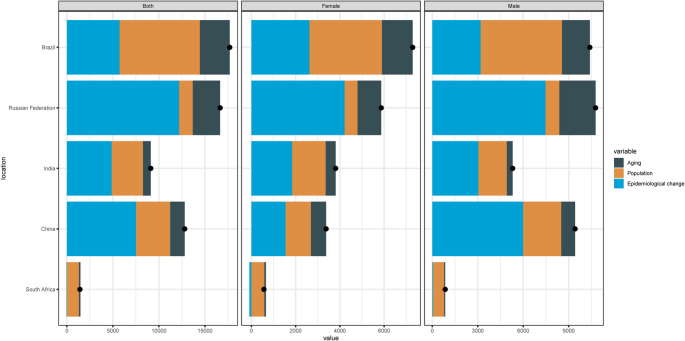
Contribution of epidemiological changes to DALY rate changes in five countries

Except for South Africa, epidemiological changes were the largest contributor to the increase in DALY rates. In Brazil, epidemiological changes contributed 30.6% and 35.9% to DALY rate changes in men and women, respectively. In India, the contributions were 57.4% in men and 48.3% in women. Similar trends were observed in China and the Russian Federation, where epidemiological changes accounted for 63.6% and 69.3% of DALY rate changes, respectively. In South Africa, population growth was the dominant factor, contributing 92.1% to DALY rate changes. In Brazil, population growth was the important factor, the contributions were 51.6% in men and 45.0% in women **(**Fig. 5**)**.

**Fig. 6:**
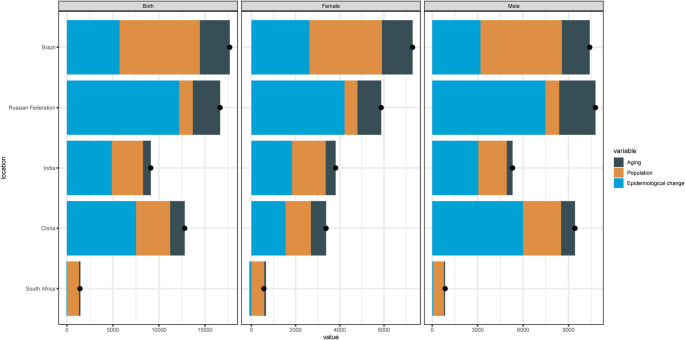
Contribution of epidemiological changes to YLL rate changes in five countries

The trends in YLL rates were consistent with those observed in DALY rates, with epidemiological changes being the primary driver in most countries. For example, in Brazil, epidemiological changes contributed 30.6% and 35.9% to YLL rate changes in men and women, respectively. In India, the contributions were 57.4% in men and 48.3% in women. In China and the Russian Federation, epidemiological changes accounted for 63.6% and 69.3% of YLL rate changes, respectively. In South Africa, population growth was the major contributor, accounting for 92.1%. Similarly, in Brazil, the contribution of the population to the change in YLL rate was 51.6% for males and 45% for females. **(**Fig. 6**)**.

### Projected burden of AA attributable to high BMI

**Fig. 7:**
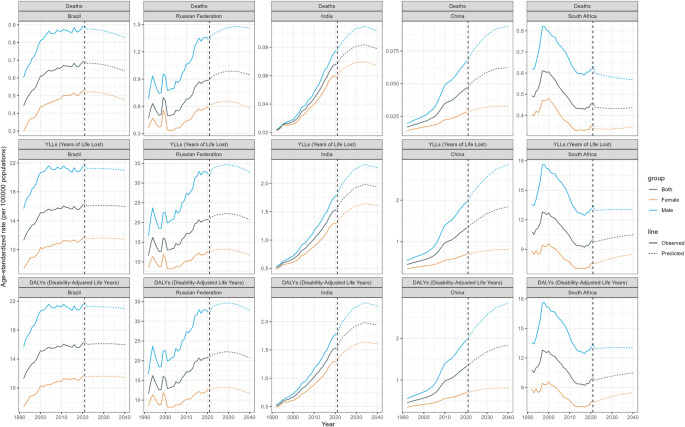
Projected Burden of AA Attributable to high BMI in five countries from 2022 to 2040

This study utilized historical data from GBD study spanning 1990 to 2021 to forecast the trends in mortality, DALY, and YLL of AA attributable to high BMI across five selected countries from 2022 to 2040. Employing BAPC model, we projected the disease burden trends over the next two decades (Table S4, Figs. 7). The results reveal significant variations in disease burden across genders and years, with an overall upward trend in most regions.

In terms of mortality rates, Brazil’s male age-standardized mortality rate is projected to decrease from 0.8766 in 2022 to 0.8296 by 2040, while female mortality rates decline from 0.5193 to 0.4740, and the overall mortality rate drops from 0.6814 to 0.6397. In the Russian Federation, male mortality rates rise from 1.3837 in 2022 to 1.4616in 2040, while female mortality rates decrease from 0.6180 to 0.5781, with the overall mortality rate increasing from 0.9216 to 0.9459. In India, male mortality rates increase from 0.0809 in 2022 to 0.0914 in 2040, and female mortality rates rise from 0.0625 to 0.0672, with the overall mortality rate climbing from 0.0714 to 0.0789. In China, male mortality rates increase from 0.0719 in 2022 to 0.0939 in 2040, and female mortality rates rise from 0.0291 to 0.0328, with the overall mortality rate increasing from 0.0497 to 0.0624. In South Africa, male mortality rates decrease from 0.6006 in 2022 to 0.5683 in 2040, while female mortality rates increase from 0.3376 to 0.3459, and the overall mortality rate slightly declines from 0.4390 to 0.4366.

Regarding YLLs, Brazil’s male YLLs decrease from 21.2223 in 2022 to 20.9837 in 2040, while female YLLs decline from 11.5051 to 11.4502, and the overall YLLs decrease from 16.0175 to 15.9885. In the Russian Federation, male YLLs decrease from 33.4760 to 32.7865, and female YLLs decline from 12.7566 to 11.7003, with the overall YLLs decreasing from 21.3621 to 20.7568. In India, male YLLs increase from 1.9050 to 2.2782 and female YLLs rise from 1.3830 to 1.6065, with the overall YLLs increasing from 1.6410 to 1.9395. In China, male YLLs increase from 2.0963 to 2.8556, and female YLLs rise from 0.7137 to 0.8116, with the overall YLLs increasing from 1.4013to 1.8413. In South Africa, male YLLs increase from 12.9425 to 13.0092, and female YLLs rise from 7.5362 to 8.4873, with the overall YLLs increasing from 9.7457 to 10.4715.

The trends in DALYs align with those of YLLs, indicating that the increase in disease burden due to high BMI is primarily driven by rising mortality rates. Overall, the disease burden in these countries shows varying degrees of increase over the coming decades, particularly among males and in specific regions. These findings provide critical insights for the formulation of public health policies, especially in resource allocation and disease prevention strategies.

## Discussion

This study harnessed data from the GBD database spanning 1990 to 2021 to examine the trends in mortality, DALYs, and YLLs due to AA associated with high BMI in five countries: Brazil, the Russian Federation, India, China, and South Africa. The findings indicate an upward trajectory in health metrics related to AA over the next two decades, particularly in mortality and years of life lost, underscoring the substantial impact of obesity on the burden of AA and highlighting the urgent need for future research and public health interventions.

In general, our results demonstrate an increasing trend in AA mortality across the five nations. For instance, China saw mortality rates rise from 0.0099 in 1990 to 0.0376 in 2021, with the Russian Federation experiencing the most significant increase, from 0.3370 in 1990 to 0.7283 in 2021. But among them, the mortality rate of AA in South Africa showed a fluctuating trend of first increasing and then decreasing. This might be related to the fact that South Africa is mainly driven by population growth, the reduction of HIV-related premature deaths among the elderly and high-risk groups, and the partial offset of risk accumulation through early medical intervention. These trends are likely linked to global lifestyle shifts and the pervasive rise in BMI, consistent with previous research indicating that AA is an escalating public health concern [3, 25]. Additionally, the analysis of YLLs and DALYs corroborates the findings related to mortality rates. Over time, these metrics have increased across all studied countries, reflecting the intensifying negative impact of AA on health span and quality of life. For example, the YLL rates in China and India are significantly higher in older age groups compared to other nations, potentially associated with the growing elderly population and the prevalence of cardiovascular risk factors in these developing countries [26]. The GBD study also found that China had the highest DALY, followed by India, Japan, the United States, and Brazil, with mortality rates closely related to the level of economic development of the country, aligning with our results [3].

We identified population aging and epidemiological transitions as the primary drivers behind the increasing burden of AA. Undoubtedly, the elderly, especially those aged 65 and above, represent a high-risk group for AA [3]. However, the evidence regarding the impact of BMI on AA is inconsistent A Swedish cohort study found no statistical association between BMI and AA [27]. Another meta-analysis reached similar conclusions [28]. Yet, numerous studies have shown that both very low (< 18.5) and high (≥ 40) BMI categories are associated with increased 30-day mortality risk in patients undergoing endovascular AA repair and open aortic repair [11, 29, 30]. Individuals with higher BMI may be more susceptible to becoming overweight or obese in the future, leading to higher variability in BMI and potentially increasing their risk of developing AA [31, 32]. AA is associated with a multitude of factors, including blood pressure and blood lipids [8]. A Mendelian randomization study found a strong positive correlation between BMI and arterial hypertension and also observed that BMI might exacerbate the risk of cardiovascular disease by influencing fat mass index [33]. Further investigation is needed to explore these mechanisms. The results from our predictive models suggest that without effective interventions, the disease burden of AA is likely to continue increasing over the coming decades.

In patients with aortic aneurysms (AA), the differences in mortality rates and DALY trends between men and women may be caused by multiple factors. At the biological level, estrogen in women may delay diseases by protecting the function of vascular endothelium. Or at the behavioral and social-cultural level, men generally have a lower attention to health management and are less likely to participate in early screenings. And national differences may be related to medical resources, the speed of aging, and public health policies. In view of the differences in the health systems among the BRICS countries, it is suggested that China strengthen the whole-process management of metabolic diseases through intelligent medical systems, Russia establish a quality control platform for vascular diseases to enhance the imaging diagnostic capabilities at the grassroots level, India implement the public-private partnership model to expand the coverage of community ultrasound screenings, South Africa integrate HIV prevention and control resources to develop a comprehensive path for chronic diseases, Brazil optimize the functions of family health stations and incorporate abdominal aortic screening into the grassroots chronic disease management packages, thus forming a precise prevention and control system based on resource allocation.

This article fills the research gap in emerging economies. The population of the BRICS countries accounts for 40% of the global population. Their disease patterns have forward-looking reference significance for developing countries and possess extremely important global health warning value. However, the GDB data relies on model estimation, and incomplete registration of grassroots information may introduce errors. In addition, the regional differences within the BRICS countries need to be further analyzed in detail. The discussion will acknowledge that while this study uses GBD 2021 estimates, the forthcoming GBD 2023 data may refine these findings once incorporated.

## Conclusions

In summary, this study underscores the necessity for targeted preventive measures. In response to the high AA burden related to BMI in the BRICS countries, a “precision prevention and control combined with system adaptation” strategy needs to be adopted: High-resource countries (such as China and Russia) focus on intelligent healthcare and specialized collaboration; Middle- and low-resource countries (such as India and South Africa) prioritize primary screening and public-private partnerships; Universal coverage countries (such as Brazil) strengthen community networks and health equity. These measures are crucial for alleviating the burden of AA over the coming decades, especially against the backdrop of rapidly aging populations and rapidly changing lifestyles. Future research should further explore the specific trends among different regions and populations to develop more targeted public health strategies.

## Electronic Supplementary Material

Below is the link to the electronic supplementary material.

**Supplementary Material 1**: **Table S1** Overall trends of AA burden attributable to high BMI in five countries from 1990 to 2021.

**Supplementary Material 2**: **Table S2** EAPC of AA burden attributable to high BMI in five countries from 1990 to 2021.

**Supplementary Material 3**: **Table S3** Decomposition analysis of AA burden attributable to high BMI in five countries.

**Supplementary Material 4**: **Table S4** Projected Burden of AA Attributable to high BMI.

## Data Availability

No datasets were generated or analysed during the current study.

## Abbreviations

AA: Aortic aneurysm
AAA: Abdominal aortic aneurysm
ASR: Age-standardized rate
BAPC: Bayesian Age-Period-Cohort
BMI: Body mass index
CI: Confidence interval
DALY: Disability-adjusted life year
EAPC: Estimated the annual percentage change
GBD: Global Burden of Disease
ICD: International Classification of Diseases
SDI: Socio-demographic Index
TAA: Thoracic aortic aneurysm
YLD: Years lived with disability
YLL: Years of life lost

## References

[1] Tyrovolas S, Tyrovola D, Gine-Vazquez I, Koyanagi A, Bernabe-Ortiz A, Rodriguez-Artalejo F, et al. Global, regional, and National burden of aortic aneurysm, 1990–2017: a systematic analysis of the global burden of disease study 2017. Eur J Prev Cardiol. 2022;29:1220–32. 10.1093/eurjpc/zwab015. 33783496 10.1093/eurjpc/zwab015 PMC11110262

[2] Huang X, Wang Z, Shen Z, Lei F, Liu YM, Chen Z, et al. Projection of global burden and risk factors for aortic aneurysm - timely warning for greater emphasis on managing blood pressure. Ann Med. 2022;54:553–64. 10.1080/07853890.2022.2034932. 35139697 10.1080/07853890.2022.2034932 PMC8843207

[3] Wang Z, You Y, Yin Z, Bao Q, Lei S, Yu J, et al. Burden of aortic aneurysm and its attributable risk factors from 1990 to 2019: an analysis of the global burden of disease study 2019. Front Cardiovasc Med. 2022;9:901225. 10.3389/fcvm.2022.901225. 35711350 10.3389/fcvm.2022.901225 PMC9197430

[4] Guirguis-Blake JM, Beil TL, Senger CA, Coppola EL. Primary care screening for abdominal aortic aneurysm: updated evidence report and systematic review for the Us preventiveservices task force. JAMA. 2019;322:2219–38. 10.1001/jama.2019.17021.31821436 10.1001/jama.2019.17021

[5] O’Gara PT. Cardiology patient page. Aortic aneurysm. Circulation. 2003;107:e43–5. 10.1161/01.cir.0000054210.62588.ed. 12591767 10.1161/01.cir.0000054210.62588.ed

[6] Roth GA, Mensah GA, Johnson CO, Addolorato G, Ammirati E, Baddour LM, et al. Global burden of cardiovascular diseases and risk factors, 1990–2019: update from the Gbd 2019 study. J Am Coll Cardiol. 2020;76:2982–3021. 10.1016/j.jacc.2020.11.010. 33309175 10.1016/j.jacc.2020.11.010 PMC7755038

[7] Zhang Y, Lai J. Spatiotemporal trends in the burden of aortic aneurysms caused by high sodium intake from 1990 to 2019: a global, regional, and National analysis. Nutr Metab Cardiovasc Dis. 2024;34:1207–16. 10.1016/j.numecd.2023.12.020. 38331643 10.1016/j.numecd.2023.12.020

[8] Gao J, Cao H, Hu G, Wu Y, Xu Y, Cui H, et al. The mechanism and therapy of aortic aneurysms. Signal Transduct Target Ther. 2023;8:55. 10.1038/s41392-023-01325-7. 36737432 10.1038/s41392-023-01325-7 PMC9898314

[9] Golledge J, Clancy P, Jamrozik K, Norman PE. Obesity, adipokines, and abdominal aortic aneurysm: health in men study. Circulation. 2007;116:2275–9. 10.1161/CIRCULATIONAHA.107.717926. 17967974 10.1161/CIRCULATIONAHA.107.717926

[10] Lu F, Lin Y, Zhou J, Chen Z, Liu Y, Zhong M, Wang L. Obesity and the obesity paradox in abdominal aortic aneurysm. Front Endocrinol (Lausanne). 2024;15:1410369. 10.3389/fendo.2024.1410369. 39055063 10.3389/fendo.2024.1410369 PMC11269098

[11] Sheng C, Liu T, Chen S, Liao M, Yang P. The neglected association between central obesity markers and abdominal aortic aneurysm presence: A systematic review and meta-analysis. Front Cardiovasc Med. 2023;10:1044560. 10.3389/fcvm.2023.1044560. 36844737 10.3389/fcvm.2023.1044560 PMC9947524

[12] Wu Y, Zhang H, Jiang D, Yin F, Guo P, Zhang X, et al. Body mass index and the risk of abdominal aortic aneurysm presence and postoperative mortality: a systematic review and dose-response meta-analysis. Int J Surg. 2024;110:2396–410. 10.1097/JS9.0000000000001125. 38320094 10.1097/JS9.0000000000001125 PMC11020033

[13] D’Oria M, Scali S, Neal D, DeMartino R, Mani K, Budtz-Lilly J, et al. The association between body mass index and death following elective endovascular and open repair of abdominal aortic aneurysms in the vascular quality initiative. Eur J Vasc Endovasc Surg. 2023;66:27–36. 10.1016/j.ejvs.2023.01.047. 36738822 10.1016/j.ejvs.2023.01.047

[14] Zhang M, Jin W, Tian Y, Zhu H, Zou N, Jia Y, et al. Cancer burden variations and convergences in globalization: a comparative study on the tracheal, bronchus, and lung (tbl) and liver cancer burdens among who regions from 1990 to 2019. J Epidemiol Glob Health. 2023;13:696–724. 10.1007/s44197-023-00144-x. 37639192 10.1007/s44197-023-00144-x PMC10686938

[15] Cao F, Xu Z, Li XX, Fu ZY, Han RY, Zhang JL, et al. Trends and cross-country inequalities in the global burden of osteoarthritis, 1990–2019: a population-based study. Ageing Res Rev. 2024;99:102382. 10.1016/j.arr.2024.102382. 38917934 10.1016/j.arr.2024.102382

[16] Liu Y, Gao Y, Yan G, Liu Y, Tian W, Zhang Y, et al. Global disease burden analysis of cardiometabolic disease attributable to second-hand smoke exposure from 1990 to 2040. Am J Prev Cardiol. 2025;21:100902. 10.1016/j.ajpc.2024.100902. 39720767 10.1016/j.ajpc.2024.100902 PMC11664086

[17] Lei S, Huang G, Li X, Xi P, Yao Z, Lin X. Global burden, trends, and inequalities of gallbladder and biliary tract cancer, 1990–2021: a decomposition and age-period-cohort analysis. Liver Int. 2025;45:e16199. 10.1111/liv.16199. 39742398 10.1111/liv.16199 PMC11688657

[18] Global incidence. prevalence, years lived with disability (ylds), disability-adjusted life-years (dalys), and healthy life expectancy (hale) for 371 diseases and injuries in 204 countries and territories and 811 subnational locations, 1990–2021: a systematic analysis for the global burden of disease study 2021. Lancet. 2024;403:2133–61. 10.1016/S0140-6736(24)00757-8. 38642570 10.1016/S0140-6736(24)00757-8 PMC11122111

[19] Hankey BF, Ries LA, Kosary CL, Feuer EJ, Merrill RM, Clegg LX, et al. Partitioning linear trends in age-adjusted rates. Cancer Causes Control. 2000;11:31–5. 10.1023/a:1008953201688. 10680727 10.1023/a:1008953201688

[20] Liu Z, Jiang Y, Yuan H, Fang Q, Cai N, Suo C, et al. The trends in incidence of primary liver cancer caused by specific etiologies: results from the global burden of disease study 2016 and implications for liver cancer prevention. J Hepatol. 2019;70:674–83. 10.1016/j.jhep.2018.12.001. 30543829 10.1016/j.jhep.2018.12.001

[21] Das GP. Standardization and decomposition of rates from cross-classified data. Genus. 1994;50:171–96. 12319256

[22] Das GP. A general method of decomposing a difference between two rates into several components. Demography. 1978;15:99–112. 631402

[23] Riebler A, Held L. Projecting the future burden of cancer: bayesian age-period-cohort analysis with integrated nested Laplace approximations. Biom J. 2017;59:531–49. 10.1002/bimj.201500263. 28139001 10.1002/bimj.201500263

[24] Jurgens V, Ess S, Cerny T, Vounatsou P. A bayesian generalized age-period-cohort power model for cancer projections. Stat Med. 2014;33:4627–36. 10.1002/sim.6248. 24996118 10.1002/sim.6248

[25] Krafcik BM, Stone DH, Cai M, Jarmel IA, Eid M, Goodney PP, et al. Changes in global mortality from aortic aneurysm. J Vasc Surg. 2024;80:81–8. 10.1016/j.jvs.2024.02.025. 38408686 10.1016/j.jvs.2024.02.025 PMC11193635

[26] Sun J, Qiao Y, Zhao M, Magnussen CG, Xi B. Global, regional, and National burden of cardiovascular diseases in youths and young adults aged 15–39 years in 204 countries/territories, 1990–2019: a systematic analysis of global burden of disease study 2019. Bmc Med. 2023;21:222. 10.1186/s12916-023-02925-4. 37365627 10.1186/s12916-023-02925-4 PMC10294522

[27] Stackelberg O, Bjorck M, Sadr-Azodi O, Larsson SC, Orsini N, Wolk A. Obesity and abdominal aortic aneurysm. Br J Surg. 2013;100:360–6. 10.1002/bjs.8983. 23203847 10.1002/bjs.8983

[28] Takagi H, Umemoto T. A meta-analysis of the association of obesity with abdominal aortic aneurysm presence. Int Angiol. 2015;34:383–91. 24945917

[29] D’Oria M, Scali S, Stone D. Response to: re. Beyond bmi: exploring body composition’s role in long term outcome of elective abdominal aortic aneurysm repair. Eur J Vasc Endovasc Surg. 2024;67:358. 10.1016/j.ejvs.2023.09.003. 37689307 10.1016/j.ejvs.2023.09.003

[30] Liang TW, Wang SK, Dimusto PD, McAninch CM, Acher CW, Timsina LR, et al. Association between body mass index and perioperative mortality after repair of ruptured abdominal aortic aneurysms. Vasc Endovascular Surg. 2020;54:573–8. 10.1177/1538574420939356. 32643559 10.1177/1538574420939356

[31] Acosta S, Fatemi S, Melander O, Engstrom G, Gottsater A. Prospective comparison of plasma biomarker and traditional risk factor profiles for incident isolated atherosclerotic disease and incident isolated abdominal aortic aneurysm. Front Cardiovasc Med. 2021;8:818656. 10.3389/fcvm.2021.818656. 35097031 10.3389/fcvm.2021.818656 PMC8790118

[32] Kato J, Kawagoe Y, Jiang D, Ida T, Shimamoto S, Igarashi K, et al. Plasma adrenomedullin level and year-by-year variability of body mass index in the general population. Peptides. 2021;142:170567. 10.1016/j.peptides.2021.170567. 33964322 10.1016/j.peptides.2021.170567

[33] Larsson SC, Back M, Rees J, Mason AM, Burgess S. Body mass index and body composition in relation to 14 cardiovascular conditions in Uk biobank: a Mendelian randomization study. Eur Heart J. 2020;41:221–6. 10.1093/eurheartj/ehz388. 31195408 10.1093/eurheartj/ehz388 PMC6945523

